# Development of Self-Consolidating High Strength Concrete Incorporating Treated Palm Oil Fuel Ash

**DOI:** 10.3390/ma8052154

**Published:** 2015-04-27

**Authors:** Belal Alsubari, Payam Shafigh, Mohd Zamin Jumaat

**Affiliations:** Department of Civil Engineering, Faculty of Engineering, University of Malaya, 50603 Kuala Lumpur, Malaysia; E-Mails: pshafigh@gmail.com (P.S.); zamin@um.edu.my (M.Z.J.)

**Keywords:** self-consolidating concrete, treated palm oil fuel ash, high volume, compressive strength, drying shrinkage, acid attack

## Abstract

Palm oil fuel ash (POFA) has previously been used as a partial cement replacement in concrete. However, limited research has been undertaken to utilize POFA in high volume in concrete. This paper presents a study on the treatment and utilization of POFA in high volume of up to 50% by weight of cement in self-consolidating high strength concrete (SCHSC). POFA was treated via heat treatment to reduce the content of unburned carbon. Ordinary Portland cement was substituted with 0%, 10%, 20%, 30%, and 50% treated POFA in SCHSC. Tests have been conducted on the fresh properties, such as filling ability, passing ability and segregation resistance, as well as compressive strength, drying shrinkage and acid attack resistance to check the effect of high volume treated POFA on SCHSC. The results revealed that compared to the control concrete mix, the fresh properties, compressive strength, drying shrinkage, and resistance against acid attack have been significantly improved. Conclusively, treated POFA can be used in high volume as a cement replacement to produce SCHSC with an improvement in its properties.

## 1. Introduction

Self-consolidating high strength concrete (SCHSC) is a new type of concrete that combines the advantages of both self-consolidating and high strength concretes. SCHSC has been developed to show good fresh properties, and exhibit high strength and excellent durability characteristics [1,2,3]. SCHSC has been used to different types of structural applications for which densely congested reinforcement concrete elements and the pumping to high levels is needed. It can be used in many applications, such as high-rise buildings, tunnel lining repairs, and congested foundations [4,5]. The cement content used in SCHSC ranges between 430 and 700 kg/m^3^ [6,7]. This leads to an increase in the cost of the structure. In addition, using high cement content in the concrete mix is a disadvantage of this type of concrete from an environmental point view. It was estimated that every one ton of cement produced 650–920 kg of CO_2_, which is approximately 7% of the global CO_2_ emissions [8,9]. This can lead to an increase in greenhouse gases and cause serious environmental problems [10].

The inclusion of supplementary cementitious materials (SCMs) in concrete, such as fly ash, silica fume, rice husk ash and palm oil fuel ash, can partially reduce the consumption of cement. This leads to a decrease in the cost of concrete, reduction in the negative environmental impact and an improvement in many engineering properties of concrete [10,11]. For example, one of the SCMs is palm oil fuel ash (POFA), which is an agro-waste produced by heating up palm oil fibers and shells to generate steam electricity in the palm oil mill [12,13]. POFA is produced annually in large amounts in some countries, such as Malaysia, Thailand and Indonesia. It was stated that, in 2007, approximately three million tons of POFA was produced in Malaysia and around 100,000 tons in Thailand [14]. This ash is landfill dumped without any beneficial returns [15]. Several studies have been conducted to check the feasibility of using ground POFA as a supplementary cementitious material (SCM) in concrete [3,12,13,16]. These studies showed that ground POFA has potential pozzolanic properties, and that it can be used to partially replace cement to enhance concrete properties. However, these studies have some limitations, such as the level of replacement should not be more than 30% and the optimum replacement level was 20% in both normal vibrated and self-compacting concretes. In addition, increasing the substitution levels to high volume reduces the early age compressive strength of concrete and increases the demand for water.

This study aimed to eliminate the limitations and disadvantages of ground POFA through heat treatment of the ground POFA to reduce the high content of unburned carbon. Thereafter, the heated POFA was ground again to obtain particles with high fineness to increase the surface area and enhance the pozzolanic reactions. The new treated POFA was used to replace the cement up to 50% by mass of Portland cement, and the fresh properties, compressive strength, drying shrinkage and acid attack of SCHSC were investigated.

## 2. Experimental Programe

### 2.1. Materials

#### 2.1.1. Cement

Ordinary Portland cement (OPC) was used in all the mixes. Some physical properties as well as the chemical compositions of the cement are shown in Table 1 and Table 2, respectively.

**Table 1:** Physical properties of aggregate, Ordinary Portland cement (OPC), ground Palm oil fuel ash (POFA), and treated POFA. BET means Brunauer–Emmett–Teller.

Property	Coarse Aggregate	Fine Aggregate	OPC	Ground POFA	Treated POFA
Maximum size (mm)	12.5	4.76	–	–	–
Water absorption (%)	0.43	1.13	–	–	–
Specific gravity	2.62	2.56	–	–	–
Fineness modulus	6.3	2.88	–	–	–
Colour	–	–	Grey	Dark grey	Grey
Passed on a 45-µm (No. 325) sieve (%)	–	–	92	96	100
Surface area, BET (m^2^/g)	–	–	–	104	–
Relative density	–	–	3.16	2.04	2.20

**Table 2:** Chemical compositions of OPC, ground POFA, and POFA (%).

Oxide Composition	OPC	Ground POFA	Treated POFA
Silicon dioxide (SiO_2_)	17.60	59.17	69.02
Aluminum trioxide (Al_2_O_3_)	4.02	3.73	3.9
Iron oxide (Fe_2_O_3_)	4.47	6.33	4.33
Calcium oxide (CaO)	67.43	5.80	5.01
Magnesium oxide (MgO)	1.33	4.87	5.18
Sodium oxide (Na_2_O)	0.03	0.18	0.18
Potassium oxide (K_2_O)	0.39	8.25	6.9
Sulfur trioxide (SO_3_)	4.18	0.72	0.41
SiO_2_ + Al_2_O_3_ + Fe_2_O_3_	–	69.23	77.25
Loss on ignition (LOI)	2.4	16.1	1.8

#### 2.1.2. Aggregate

Local mining sand and crushed granite were used as fine and coarse aggregates, respectively. Some physical properties of the aggregates are shown in Table 1.

#### 2.1.3. Preparation of Palm Oil Fuel Ash

The original POFA was collected from a local palm oil mill in Selangor, Malaysia. First, it was dried in an oven at a temperature of 105 °C for 24 h. Then, the dried POFA was sieved using a 300 µm sieve to remove larger sized particles. A Los Angeles machine was used to grind the POFA to achieve finer particles. Thirty mild steel-rods of 10 mm diameter and 500 mm length were placed into the rotating drum together with about 6 kg of ash. For grinding the raw POFA, the drum was set to rotate for 18 h using an electric motor at a speed of 33.3 rpm. Then, the ground POFA was burnt at a temperature of 600 °C for 2 h using an electric furnace to reduce the LOI content. After that, the treated POFA was ground again to obtain finer particles.

### 2.2. Mix Proportions and Testing Methods

The treated POFA was used as a cement replacement at different percentages of 10%, 20%, 30% and 50% by mass of total binder. The mix proportions of all the mixes are shown in Table 3. All the mixes had the same binder content of 480 kg/m^3^. The water/binder ratio was kept constant at 0.35, and the coarse aggregate percentage to total aggregate was 45% for all the mixes. A superplasticizer (SP) based on an aqueous solution of modified polycarboxylate copolymers was used in the concrete mixes to achieve the requirements for the fresh properties of SCHSC.

**Table 3:** Mix proportions of concretes.

Mix No.	Cement (kg/m^3^)	Water (kg/m^3^)	*W*/*C* Ratio	POFA (kg/m^3^)	POFA (%)	Fine Aggregate (kg/m^3^)	Coarse Aggregate (kg/m^3^)	Superplasticizer (S.P) (% B)
SCHSC0	480	168	0.35	0	0	925	758.2	1.3
SCHSC10	432	168	0.35	48	10	923	752	1.3
SCHSC20	384	168	0.35	96	20	948	772	1.3
SCHSC30	336	168	0.35	144	30	944	772	1.3
SCHSC50	240	168	0.35	240	50	896	728	1.3

The physical properties and chemical compositions of POFA before and after treatment were investigated. The colour of POFA was visually inspected. The BET surface area test was conducted to check the fineness of the POFA before and after treatment. The chemical composition and LOI test were checked using the X-ray Fluorescence (XRF) technique. A Field Emission Scanning Electron Microscope (FESEM) and Energy Dispersive X-Ray Analysis (EDX) were also used to evaluate the changes in the physical properties and compositions of the POFA due to the heat treatment and the grinding process.

The fresh properties of the mixes were investigated immediately after mixing. A slump flow and *T*_50cm_ tests were conducted first. If the mix fulfilled both tests, it was then subjected to the V-funnel, J-ring, L-box, and segregation resistance tests. All the fresh properties were conducted according to European guidelines for self-compacting concrete (EFNARC) 2002 [17].

To check the compressive strength of concrete at the ages of 1, 3, 7, 28, 56, and 90 days, 100 mm cubes were tested according to BS EN 12390-3:2002 [18]. A compression-testing machine of 3000 kN capacity was used to test three specimens for each test.

The drying shrinkage strain of concrete was determined by using prismatic concrete specimens of 100 × 100 × 500 m^3^. These specimens were demoulded 24 h after casting, and cured in water for seven days. Then, the specimens were removed from the water and wiped with a damp cloth. Thereafter, demec points were fitted to three sides of each specimen. The distance between two demec points was 200 mm. The concrete specimens were then kept in a room with a temperature of 28 ± 3 °C and a relative humidity of 74% ± 4%.

The acid attack test was conducted to check the resistance of the concrete specimens to chemical attacks. After seven days moist curing, the specimens were immersed in a 3% hydrochloric acid (HCL) solution with a pH of about two. The specimens were immersed in 3% HCL acid solution for 1800 h (75 days). This solution was checked at regular intervals of two weeks to maintain a constant concentration during the test period. The compressive strength and mass of the concrete specimens were measured after 1800 h.

## 3. Results and Discussion

### 3.1. Physical Characteristics and Chemical Compositions of Treated POFA

**Figure 1:**
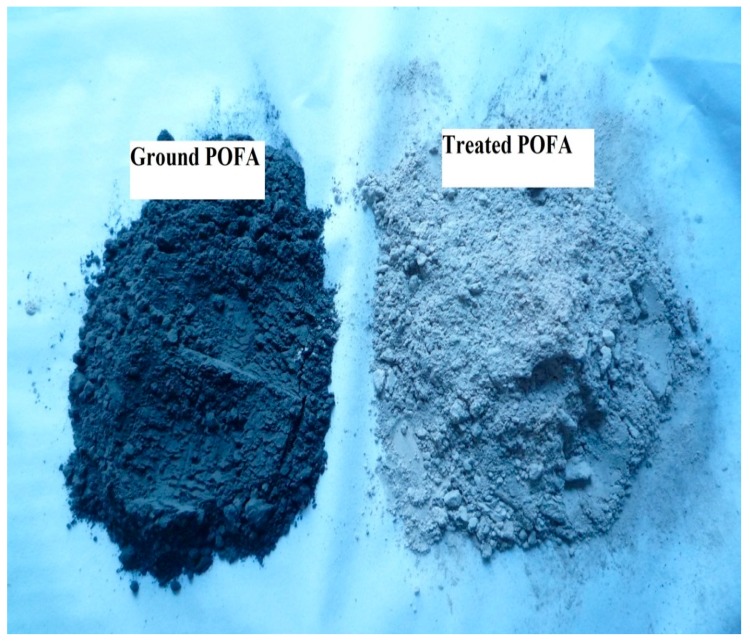
Difference in colour for ground POFA and treated POFA.

**Figure 2:**
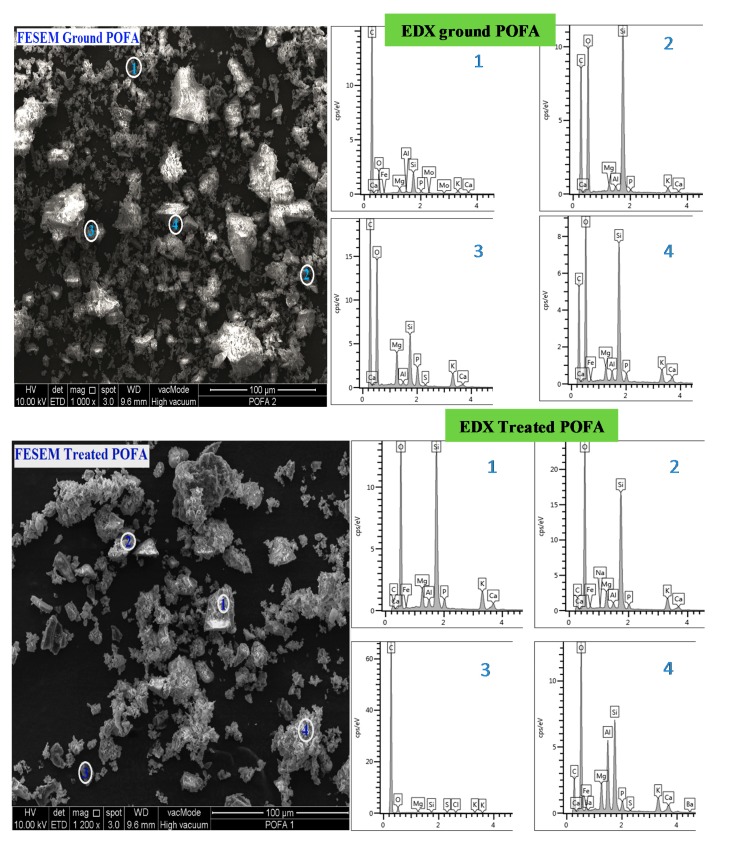
FESEM for ground POFA and treated POFA.

The physical properties and chemical composition of OPC, ground POFA, and treated POFA are shown in Table 1 and Table 2, respectively. After the treatment process, the colour of the POFA had changed from dark grey to grey, as shown in Figure 1. This can be attributed to the removal of unburned carbon through the heating treatment. The FESEM and EDX tests for ground POFA and treated POFA showed an improvement in the size, the surface area and the chemical composition of the treated POFA, as shown in Figure 2. The overall chemical properties of the POFA were improved, such as the increase in the content of the silica dioxide (SiO_2_), which is the main component of POFA, from 59% to 66%. Hence, the sum of SiO2, Fe_2_O_3_, and Al_2_O_3_ increased from 69% to 77% due to the reduction in the carbon content. In addition, one of the benefits of using the heat treatment for POFA is the significant reduction of the LOI content, which reduced from 16.1% to 1.8%. The surface area of the POFA increased from 4.9 m^2^/g for ground POFA to 7.4 m^2^/g (about 52%) for treated POFA due to the heat treatment and the second grinding process.

### 3.2. Fresh Properties

To fulfil the requirement for fresh self-consolidating high strength concrete (SCC), fresh properties tests were conducted according to EFNARC [17]. The fresh concrete was evaluated by conducting the filling ability, passing ability, and segregation resistance tests.

#### 3.2.1. Filling Ability

Filling ability refers to the ability of concrete to flow horizontally and reach all the corners of a formwork under its self-weight of concrete without vibration [17]. The filling ability of the different mixes of SCHSC was examined with respect to the slump flow, *T*_50cm_ spread time, and V-funnel flow time.

##### Slump Flow and T_50_cm Spread Time

**Figure 3:**
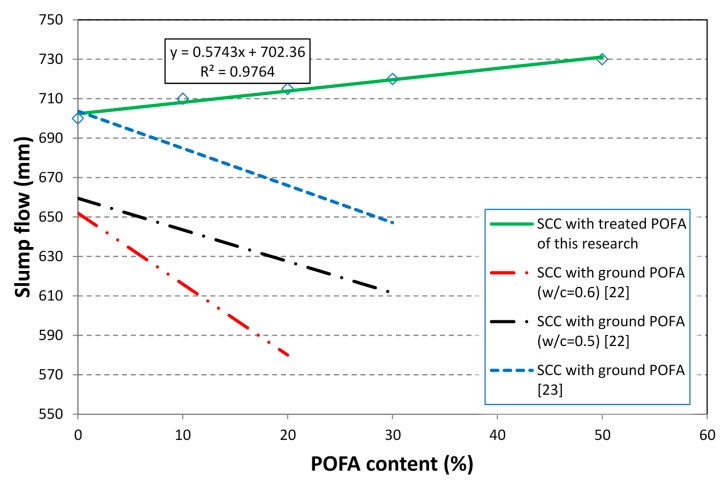
Slump flow for SCC with ground POFA and treated POFA.

**Figure 4:**
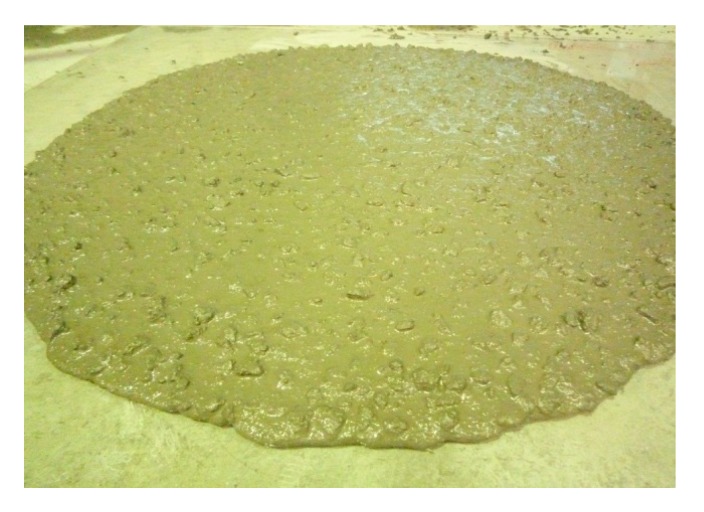
Visual appearance of concrete containing 50% treated POFA during slump flow test.

The slump flows of the mixes were determined using an Abram’s slump cone according to EFNARC guidelines 2002. All mixes were designed for a slump flow of average diameter of 710 ± 20 mm. For all the mixes, the slump flow was in the range of 700–730 mm, which is in agreement with the EFNARC standard [17]. As shown in Figure 3, it can be seen that the mixes incorporating the treated POFA could enhance the slump flow. Figure 4 shows the flow of the mix containing 50% treated POFA. As can be seen in this figure, this mix has a good flow and cohesive mix compositions. No bleeding or segregation can be observed. The improvement of the fresh properties can be attributed to the reduction of the unburned carbon content of the treated POFA. In addition, the improvement of the fresh properties of SCHSC containing the treated POFA is the higher binder volume in the concrete. This is because the specific gravity of the treated POFA is less than OPC.

**Figure 5:**
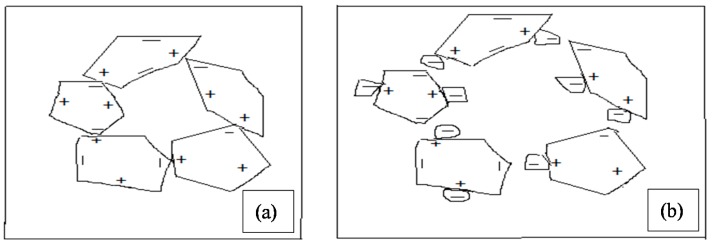
Diagrammatic representation of treated POFA particles adsorbs on and disperses cement particles; (**a**) Flocculation caused by oppositely (**b**) Treated POFA particles adsorb on cement charged surfaces of cement particles Surfaces of cement particles resulting in dispersion opposite charges of cement.

Another possible reason, as shown in Figure 5a,b, the fine particles of the treated POFA are adsorbed on the oppositely charged surfaces of cement particles and prevent them from flocculation. Thus, the cement particles are dispersed effectively and will not trap large amounts of water. Similar findings on high-performance concrete containing high-volume fly ash were reported by Malhotra *et al.* [11]. However, several previous studies reported that one of the challenges faced is the reduction of concrete workability as the POFA content increases [15,19,20,21]. In addition, a recent research conducted on the self-compacting concrete containing ground POFA up to 20% cement replacement showed that, as the percentage of replacement increased, the slump flow of concrete decreased, as shown in Figure 3 [22,23]. This can be attributed to the higher content of unburned carbon, which absorbs more SP than the other particles [14,24]. In addition, the time taken for concrete to reach 50 cm after lifting the slump cone (*T*_50cm_) was measured at the mean time of conducting the slump flow. As can be seen in Table 4, as the treated POFA content increased, the *T*_50cm_ time decreased. The lower *T*_50cm_ indicates good filling ability. However, researchers have reported that as the level of replacement is high, the *T*_50cm_ increased due to the increase in the viscosity of SCC [22,25]. The obtained results for *T*_50cm_ are in agreement with the findings on the self-compacting concrete containing fly ash up to 50% replacement [26]. From these tests, it can be concluded that the treated POFA significantly improved the workability in terms of slump flow and *T*_50cm_ compared to ground POFA.

**Table 4:** Fresh properties of concrete.

Mix No.	Filling Ability			Passing Ability			Segregation Resistance
	Slump Flow (mm)	*T*_50_ Spread Time (s)	V-Funnel Time (s)	J-Ring (mm)		L-Box	Segregation Index (%)
				Difference in Heights (mm)	Flow (mm)		
SCHSC0	700	3.5	6.6	9	690	94.0	6.7
SCHSC10	710	3.4	6.4	8	690	95.0	6.9
SCHSC20	715	3.2	6.0	7	695	97.0	7.3
SCHSC30	720	3.0	5.9	7	700	1.0	7.8
SCHSC50	730	2.9	5.8	5	710	1.0	8.2

##### V-Funnel Test

**Figure 6:**
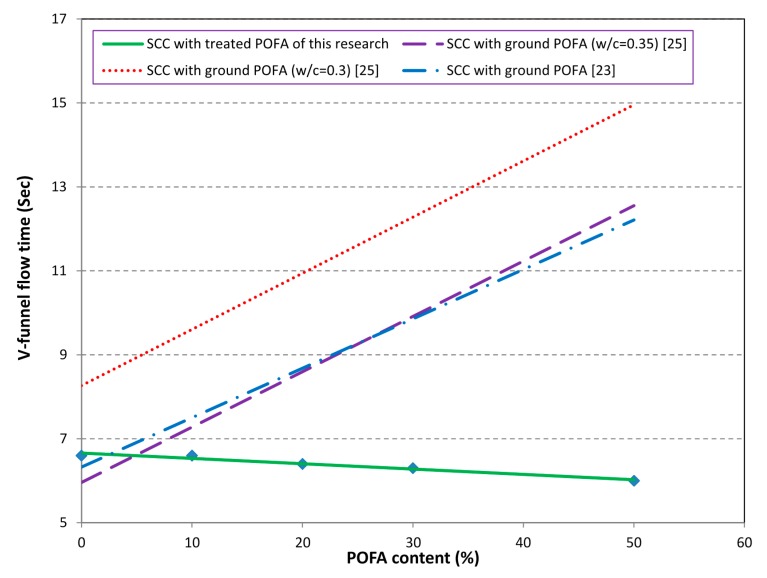
V-funnel for SCC with ground POFA and treated POFA.

The V-funnel test was conducted to measure the filling ability of the mixes. The values of V-funnel vary in the range of 6.0–6.6 s. These values are considered to be appropriate and fulfill the requirement of EFNARC [17]. It can be seen from Figure 6 that in contrast to ground POFA, as the content of the treated POFA increased, the V-funnel time decreased linearly. The shorter flow times indicate greater flowability. This improvement can be attributed to the low plastic viscosity of the mixes containing the treated POFA. However, previous research reported that as the ground POFA content increased, the V-funnel time was also increased due to the higher viscosity and yield value [22,23,25]. The results obtained from this study were in agreement with previous studies on self-compacting concrete containing fly ash up to 50% replacement [26].

#### 3.2.2. Passing Ability

Passing ability can be defined as the ability of SCC to pass congested reinforcement and small openings under its self-weight without vibration. This was evaluated by conducting J-ring and L-box tests.

##### J-Ring Test

**Figure 7:**
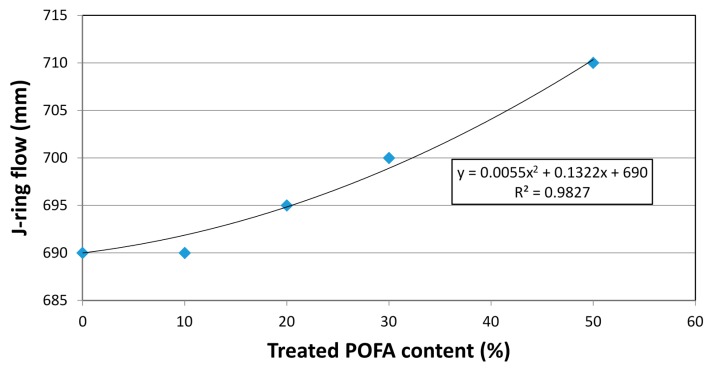
J-ring flow for SCC with treated POFA.

The J-ring test was measured with respect to the difference between the height of the concrete inside and outside of J-ring bars; the diameter of the concrete was also measured. The differences in height of the different mixes varied in the range of 6–9 mm, as shown in Table 4. The concrete mix containing 50% treated POFA (SCHSC50) showed the lowest value (6 mm) due to the lower viscosity and shear stress, which allowed the concrete to flow more freely. In addition, the diameter of the concrete was measured, and the results showed that the SCHSC50 exhibited the higher diameter of concrete containing treated POFA due to the lower specific gravity compared to concrete made with OPC, as shown in Figure 7. The results obtained are in agreement with previous finding reported by Johari *et al.* [14]. Safiuddin *et al.* [22] and Alsubari *et al.* [23] have reported that as the content of ground POFA increased, the J-ring flow was decreased. This is due to the nature of the ground POFA containing unburned carbon, which absorbs more water and SP, which leads to a decrease in the workability of the concrete [14,24].

##### L-Box

**Figure 8:**
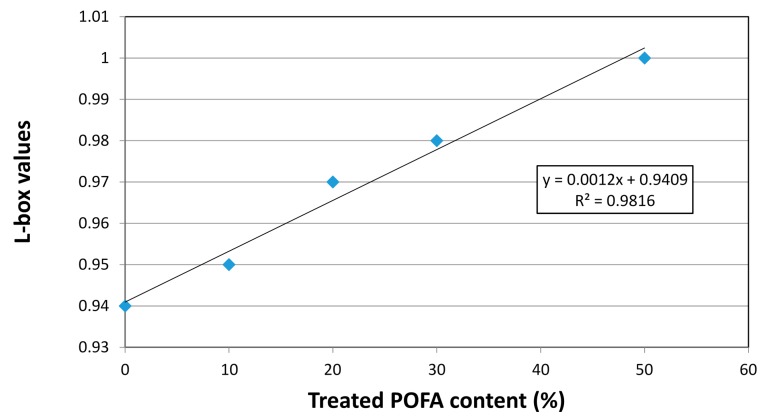
L-box for SCC with treated POFA.

The L-box test is normally used to assess the passing ability of SCC when it is subjected to reinforcement blocks [17]. The results of the L-box of concretes containing 0%, 10%, 20%, 30%, and 50% were 0.94, 0.95, 0.97, 0.98, and 1.0, respectively. The test results showed that as the treated POFA content increased, the value of the L-box also increased, as shown in Figure 8. This can be attributed to the lower viscosity and yield value of concrete containing the treated POFA.

#### 3.2.3. Segregation Resistance

**Figure 9:**
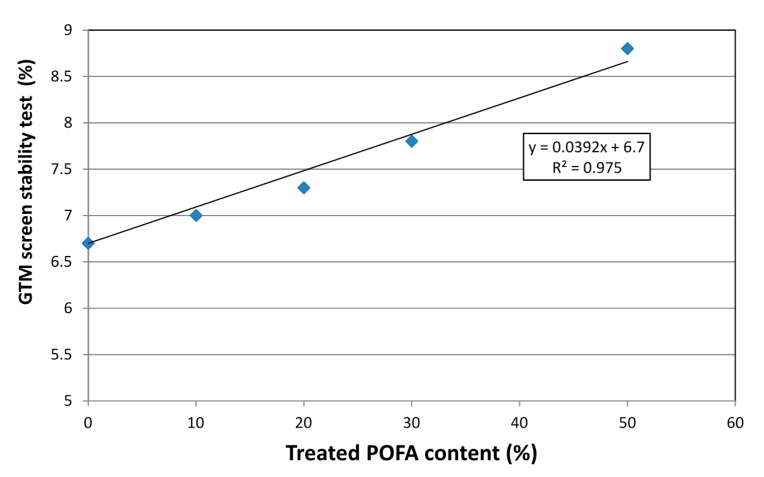
Segregation resistance for SCC with treated POFA.

Segregation resistance is a very important factor in SCC. The mix compositions must remain homogeneous and uniform during and after the process of transport and placing [17,27]. Furthermore, it should be ensured that all the aggregate is relatively equivalent at all locations and at all levels to avoid any deformability and blocking [28]. All the mixes were visually inspected during the slump flow, J-ring, and L-box tests. It was observed that there was no segregation or bleeding in any of the mixes. In addition, the GTM screen stability test was conducted to confirm the visual observations for segregation resistance. As can be seen from Figure 9, the GTM screen stability values for concretes containing 0%, 10%, 20%, 30%, and 50% were 6.7%, 7.0%, 7.3%, 7.8%, and 8.8%, respectively. According to EFNARC [17], the values of GTM should not be more than 15%. The results obtained showed good segregation resistance and all the values were in the range specified in the EFNARC guidelines [17]. It can be seen that by increasing the substitution level the segregation value increased. This is because the substitution was by mass, therefore, the volume of paste of SCHSC50 was higher than SCHSC0, resulting in a higher mortar volume in the mix, which leads to an increase in the segregation. In addition, the higher flow and lower viscosity of concrete containing the treated POFA lead to an increase in the mortar passing through the GTM sieve. The results confirmed the previous tests for slump flow, J-ring, and L-box.

### 3.3. Compressive Strength

**Figure 10:**
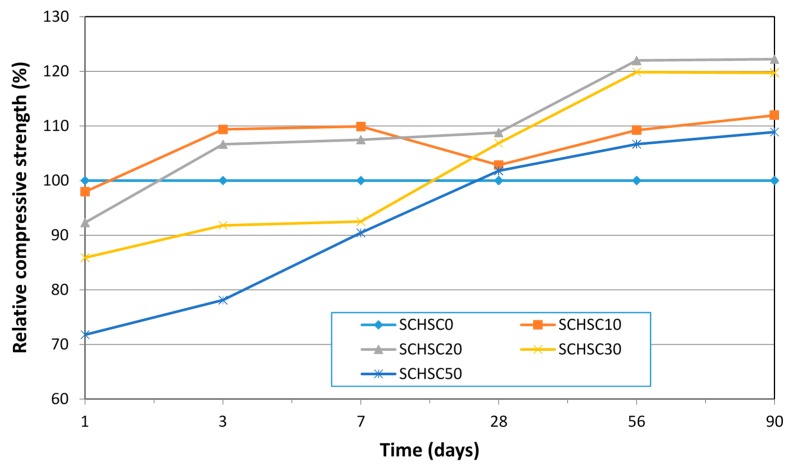
Relative compressive strengths of SCHSCs with different treatment of POFA content.

The compressive strength developments and the relative compressive strength of the different mixes are shown in Table 5. All the concrete mixes containing the treated POFA showed high early strength at one day, which is adequate for formwork removal. Malhotra *et al.* [11] have reported that the required compressive strength at one day for removing formwork is between 8 and 10 MPa. At the early ages of three and seven days, the compressive strength of concretes containing 10% and 20% cement replacement by the treated POFA showed slightly improvement of about 9% and 7% at three days, and 10% and 8% at seven days curing, respectively. Whereas, there was a reduction in the compressive strength at 30% and 50% replacement levels of about 9% and 21% at three days, and 7% and 10% at seven days, respectively. As shown in Figure 10, the improvement at the early age compressive strength for concretes containing 10% and 20% treated POFA can be mainly attributed to the high fineness of the treated POFA, which acts as a micro-filler between the particles of cement and fills the voids and improves the microstructure of the concrete [15,29], while, at higher replacement levels (30% and 50%), it showed a reduction in compressive strength. The reduction in compressive strength is mainly due to the huge reduction in the cement content [14,30]. Similar results for the self-compacting concrete and normally vibrated concrete containing rice husk ash (RHA) showed that the early age compressive strength for 10% and 20% cement replacement was higher than the control concrete due to the higher fineness of RHA [26,31].

The compressive strength of concretes at later ages containing the treated POFA showed higher compressive strength compared to the concrete made with OPC only. At 28 days, the compressive strength for mixes SCHSC0, SCHSC10, SCHSC20, SCHSC30, and SCHSC50 were found to be 67, 72, 74, 70, and 69 MPa, respectively. All the concrete mixes showed compressive strength more than 60MPa after curing for 28 days. Thus, all the mixes could be classified as self-consolidating high strength concrete. As shown in Table 5, with curing continuing up to 90 days, the strength of the concrete containing treated POFA still increased and the percentage of increase was found to be about 20%, 31%, 28%, and 16% for mixes SCHSC10, SCHSC20, SCHSC30, and SCHSC50, respectively, in comparison to the 28-day control concrete. There are two mechanisms to explain the improvement in the compressive strength of concrete containing the treated POFA. The first mechanism is the micro-filler effect of the treated POFA, which contributed to the early age strength by filling the voids between the particles, and improved the overall strength of the concrete. The second mechanism is the pozzolanic reaction of the treated POFA with high fineness and a high surface area. The silica dioxide of the treated POFA reacts chemically with the calcium hydroxide (Ca(OH_2_)) to form a secondary calcium-silicate-hydrate (C-S-H), which improves the interfacial bonds between the paste and the aggregate, as well as densifies the microstructure of the concrete. The treated POFA showed that it can be used in a high level of replacement with improvement in the compressive strength of the concrete; whereas previous research [25,32,33,34] on concrete containing ground POFA reported that the optimum replacement percentage by the ground POFA is between 10% and 20%, and that when the level of replacement is more than 30% it reduced the compressive strength of concrete, especially at early ages. The results from this research are in agreement with the findings on vibrated high strength concrete containing treated POFA reported by Johari. *et al.* [14]. In addition, the results from this study are in agreement with a previous study on self-compacting concrete containing fly ash up to 50% replacement [26].

**Table 5:** Compressive strength and the relative compressive strength for SCHSCs.

Sample No.	1 Day		3 Days		7 Days		28 Days		56 Days		90 Days	
	(MPa)	*R*_1/28c_ * (%)	(MPa)	*R*_3/28c_ * (%)	(MPa)	*R*_7/28c_ (%)	(MPa)	*R*_28/28c_ (%)	(MPa)	*R*_56/28c_ (%)	(MPa)	*R*_90/28c_ (%)
SCHSC0	39.0	0.58	51.2	0.76	57.5	0.85	67.1	1.00	70.5	1.04	72.0	1.07
SCHSC10	38.2	0.56	56.0	0.83	63.2	0.94	69.0	1.02	77.0	1.15	80.6	1.20
SCHSC20	36.0	0.53	54.6	0.80	61.8	0.91	73.0	1.09	86.0	1.28	88.0	1.31
SCHSC30	33.5	0.49	47.0	0.70	53.2	0.80	71.7	1.07	84.5	1.25	86.2	1.28
SCHSC50	28.0	0.41	40.0	0.60	52.0	0.78	68.3	1.01	75.2	1.12	78.4	1.16

* *R*_1/28c_, *R*_3/28c_, *R*_7/28c_, *R*_28/28c_, *R*_56/28c_, *R*_90/28c_ are the relative compressive strengths compared to 28 day compressive for control concrete.

### 3.4. Drying Shrinkage Strain

**Figure 11:**
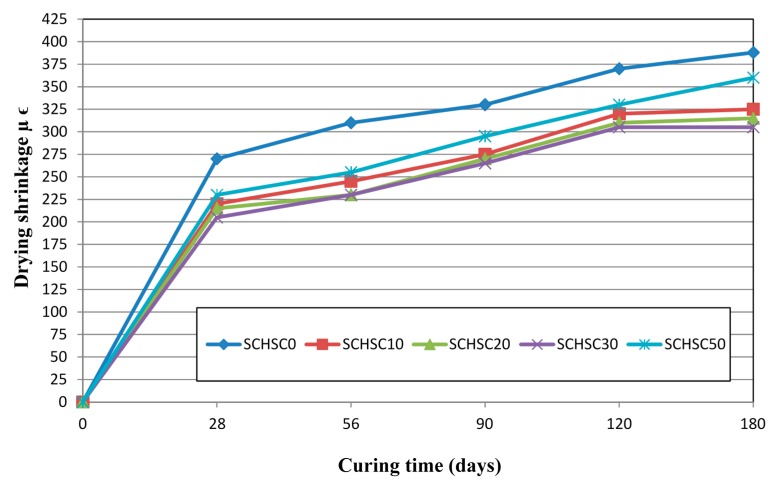
Development of drying shrinkage strain with time.

Drying shrinkage happens when water evaporates from the concrete, and mainly occurs at the early age of the concrete. As can be seen in Figure 11, all the concrete mixes containing the treated POFA exhibited lower drying shrinkage compared to concrete made with Portland cement. After 180 days, the drying shrinkage of SCHSC0, SCHSC10, SCHSC20, SCHSC30, and SCHSC50 mixes were found to be 370, 320, 310, 305, and 330 microstrains, respectively. Compared to the control mix, the reduction for mixes SCHSC10, SCHSC20, SCHSC30, and SCHSC50 were found to be 14%, 16%, 17%, and 11%, respectively. The improvement in the drying shrinkage could be attributed to the densification and enhancement of the concrete containing treated POFA due to the pozzolanic reaction. In addition, the high fineness of the treated POFA filled the voids resulting in denser concrete [3,35]. Furthermore, another possible reason is the high compaction of SCHSC, which reduces the porosity and compensates the negative effect of the reduction of the coarse aggregate [1]. Tangchirapat *et al.* [3] reported that high strength concrete containing ground POFA up to 30% cement replacement showed lower drying shrinkage in comparison with concrete made with OPC only. In addition, Mahmud *et al.* [36], and Malhotra *et al.* [11] reported that the use of supplementary cementitious materials (SCMs), such as rice husk ash (RHA) and fly ash (FA) reduced the drying shrinkage when compared with concrete made of OPC only. This can be mainly attributed to the lower cement content when SCMs are used in concrete.

### 3.5. Acid Attack

The chemical resistance of the concretes containing the treated POFA was evaluated through chemical attack by immersing 100 mm cubes in 3% hydrochloric acid solution for 1800 h. Compressive strength reduction, mass loss, change in colour, and corner losses were investigated.

#### 3.5.1. Reduction in Compressive Strength

**Figure 12:**
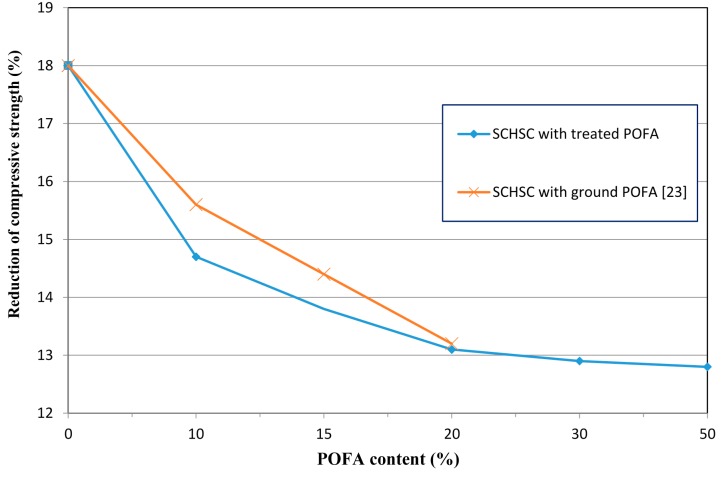
Relationship between reduction in compressive strength and treated POFA content.

Figure 12 shows the relationship between the treated POFA content and the compressive strength loss of concrete. All the concrete mixes containing treated POFA showed lower compressive strength reduction compared to concrete made with OPC only. The reduction in compressive strength of SCHSC0, SCHSC10, SCHSC20, SCHSC30, SCHSC50 were about 18%, 14.7%, 13.0%, 12.5%, and 12.0%, respectively. The improvement in the specimens containing the treated POFA could be attributed to the pozzolanic reaction of POFA with the free lime Ca(OH)_2_ to produce more C–S–H gel, leading to denser and less porous concrete. This process reduces the amount of Ca(OH)_2_, which is considered to be a very weak product against chemical attack, and enhances the concrete resistance against acid attack. In addition, the fineness of the ash led to the voids filling up and reducing the porosity of the concrete, which reduced the penetration of the acid solution into the interior parts of the concrete. A recent study on SCHSC by Alsubari *et al.* [23] reported that the use of ground POFA up to 20% cement replacement improved the concrete against acid attack, as shown in Figure 10. The treated POFA showed better resistance than ground POFA due to the higher percentage of silica and finer particles. Similar conclusions were reported by Budiea *et al.* [37] and Hussin *et al.* [38] on normal vibrated high strength and high performance concrete containing ground POFA. They concluded that the use of ground POFA in concrete showed better resistance against aggressive acids due to the pozzolanic reaction. In addition, Dinakar *et al.* and Verma *et al.* [39,40] investigated the use of fly ash in self-compacting and normally vibrated concretes exposed to acid attack. They found that concrete containing fly ash exhibited better resistance against acid attack than plain concrete. They concluded that this is due to the low amount of reaction compounds like calcium hydroxide (Ca(OH)_2_) at a lower level of cement content for the deterioration process.

#### 3.5.2. Mass Loss

**Figure 13:**
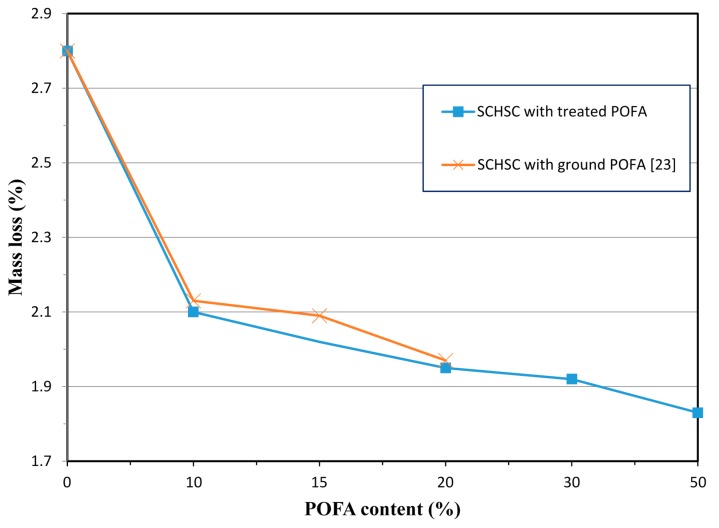
Relationship between mass loss and treated POFA content.

Mass loss was measured after immersion of the specimens in 3% HCL after 1800 h. As can be seen in Figure 11, the specimens integrated with the treated POFA exhibited lower mass loss compared to the control specimens. The results of the weight loss of the mixes SCHSC0, SCHSC10, SCHSC20, SCHSC30, and SCHSC50 were 2.8%, 2.1%, 1.95%, 1.92, and 1.84%, respectively. The higher mass loss of the control specimens is mainly due to the higher deterioration at the corners and edges, while the specimens containing the treated POFA only suffered from minor surface erosion and less corner loss, as shown in Figure 13. The concrete containing treated POFA showed better resistance against acid attack. The results obtained in this research match a previous study on self-consolidating high strength concrete containing ground POFA [23]. The high resistance against the acid attack of SCHSC incorporating treated and ground POFA was attributed to the pozzolanic reaction of the POFA conversion of calcium hydroxide (Ca(OH)_2_) to the additional C-S-H gel. Therefore, the Ca(OH)_2_, which is considered to be the weakest product from cement hydration and is highly susceptible to chemical attacks, will be reduced. This result is in line with the findings reported by Siad *et al.* [41] in the case of self-compacting concrete (SCC) with natural pozzolan. He concluded that the inclusion of natural pozzolan improved SCC against acid attack.

#### 3.5.3. Visual Inspections

**Figure 14:**
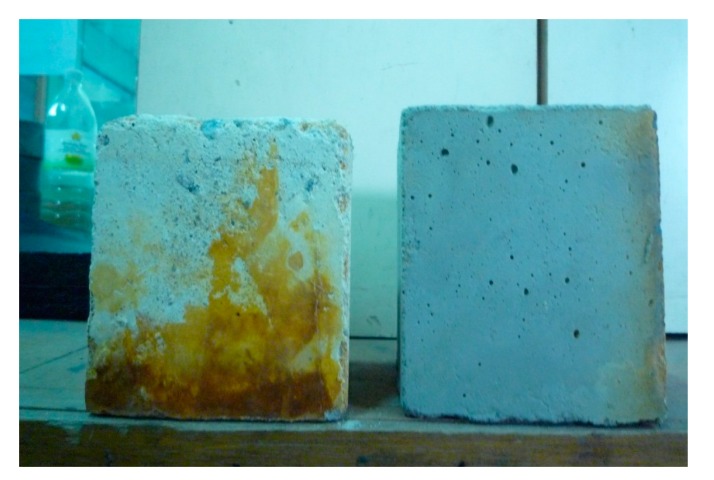
Change in colour and corner losses for SCHSC with and without treated POFA.

In addition, visual observations were conducted to check the difference in colour and corner losses of specimens immersed in acid. From Figure 14, it can be seen that the SCHSC0 samples exhibited more changes in colour and corner losses due to the effect of the acid. However, there was not much change in colour and corner mass loss for the SCHSC containing the treated POFA. This result matches the findings for self-consolidating high strength concrete and normally vibrated high strength concretes containing ground POFA reported by Alsubari *et al.* [23] and Budiea *et al.* [37]. They found that concrete containing ground POFA has less corners losses and not much change in the colours on the faces of the specimens.

## 4. Conclusions

Based on the results obtained from this study, the following conclusions can be drawn:

1. The physical properties and the chemical compositions of POFA were significantly improved via heat treatment and the grinding process.
2. Treated POFA can be utilized in higher percentage (up to 50%) with an improvement in the concrete properties compared to ground POFA.
3. SCHSCs containing treated POFA exhibited better fresh properties than the control mix.
4. Incorporating treated POFA up to 50% cement replacement in SCHSCs showed higher compressive strength compared to concrete made with OPC at 28 days of water curing.
5. Incorporating treated POFA caused a reduction in the drying shrinkage strain of all the mixes containing treated POFA compared to concrete just made with OPC.
6. The specimens containing treated POFA showed better resistance against hydrochloric acid solution compared to SCHSC only made with OPC.
